# In vitro synergy of antibiotic combinations against planktonic and biofilm Pseudomonas aeruginosa

**DOI:** 10.3205/dgkh000302

**Published:** 2017-10-17

**Authors:** Hossein Ghorbani, Mohammad Yousef Memar, Fatemeh Yeganeh Sefidan, Mina Yekani, Reza Ghotaslou

**Affiliations:** 1 Infectious and Tropical Diseases Research Center, Tabriz University of Medical Sciences, Tabriz, Iran; 2 Department of Microbiology, School of Medicine, Tabriz University of Medical Sciences, Tabriz, Iran; 3 Students’ Research Committee, Tabriz University of Medical Sciences, Tabriz, Iran

**Keywords:** antibiofilm, combination therapy, Pseudomonas aeruginosa, synergetic effects

## Abstract

**Aim:** The combination of different antimicrobial agents and subsequent synergetic effects may be beneficial in treatment of *P. aeruginosa* infections. The aim of the present study was to determine antibiotic susceptibility patterns of clinical isolates of *P. aeruginosa* and the effect of different antibiotic combinations against the multidrug-resistant (MDR), biofilm-producing bacterium *P. aeruginosa*.

**Methods:** Thirty-six *P. aeruginosa* clinical isolates were evaluated. The disk diffusion method was performed to determine antibiotic susceptibility patterns according to the Clinical and Laboratory Standards Institute (CLSI) guidelines. The minimum inhibitory concentration of antimicrobial agents for the test organisms was determined by the broth microdilution method. To determine synergetic effects of the combinations of agents, the checkerboard assay and the fractional inhibitory concentration were used. The biofilm inhibitory concentration was determined to detect any inhibitory effect of antibiotics against the biofilm.

**Results:** High levels of resistance were detected against most antibiotics, except colistin and polymyxin. According to the disk diffusion method, 58.3% of isolates were MDR. A synergetic effect between amikacin/ceftazidime, tobramycin/colistin and ceftazidime/colistin was found in 55.6%, 58.3% and 52.8% of isolates, respectively. A significant synergetic effect against biofilm-producing isolates was observed for the combination of tobramycin (0.5–1 µg/ml) and clarithromycin (256–512 µg/ml).

**Conclusion:** Combinations of antibiotics have a different activity on the biofilm and planktonic forms of *P. aeruginosa*. Consequently, separate detection of antibacterial and antibiofilm effects of the antibiotic combinations may be useful in guiding the antibiotic therapy.

## Zusammenfassung

**Zielsetzung:** Die Kombination verschiedener Antibiotika zur Erzielung eines Synergismus kann vorteilhaft zur Behandlung von *P. aeruginosa*-Infektionen sein. Ziel der Studie war die Bestimmung der Antibiotikaempfindlichkeit klinischer *P. aeruginosa*-Isolate und die Wirkung verschiedener Antibiotikakombinationen gegen multiresistente und biofilmbildene *P. aeruginosa*-Stämme

**Methode:** Es wurden 36 klinische *P. aeruginosa*-Isolate untersucht. Zur Bestimmung der Antibiotikaempfindlichkeit wurde die Plättchendiffusionsmethode gemäß der Richtlinie des Clinical and Laboratory Standards Institute (CLSI) eingesetzt. Die minimale Hemmkonzentration der Antibiotika wurde mit der Bouillon-Mikroverdünnungsmethode bestimmt. Zur Bestimmung synergistischer Kombinationseffekte wurde der Checkerboard Assay durchgeführt und die fraktionierte Hemmkonzentration ermittelt. Die Biofilm-Hemmkonzentration der Antibiotika wurde gegen in Mikrotiterplatten kultivierte Biofilme ermittelt.

**Ergebnisse: **Außer bei Colistin und Polymyxin war bei den Antibiotika eine High-Level-Resistenz nachweisbar, wobei 58,3% der Isolate multiresistent waren. Synergismus wurde für die Kombinationen Amikacin/Ceftazidim, Tobramycin/Colistin und Ceftazidim/Colistin in 55,6%, 58,3% bzw. 52,8% der Isolate nachgewiesen. Gegen Biofilm bildende Isolate wurde eine signifikante synergistische Wirkung für die Kombination Tobramycin (0.5–1 µg/ml) und Clarithromycin (256–512 µg/ml) nachgewiesen.

**Schlussfolgerung:** Die Kombination von je zwei Antibiotika hat unterschiedliche Wirkungen gegen biofilmbildende und planktonische *P. aeruginosa-*Isolate. Daher kann die separate Bestimmung der antibakteriellen und Antibiofilmwirksamkeit von Antibiotikakombinationen für die antibiotische Therapie hilfreich sein.

## Introduction

Antimicrobial resistance has emerged as a significant challenge in the treatment of infections [1]. Biofilms, which can be described as surface-attached layers of microbial cells with self-produced extracellular polymeric compounds, are a critical source of morbidity and mortality in medical practice. Biofilms may develop on human tissues as well as on a diversity of surfaces, such as prosthetic devices, venous catheters, and cardiac pacemakers; their management can be complicated and costly because they are often intrinsically resistant to high levels of antimicrobial drugs [2]. *Pseudomonas aeruginosa* is accepted as a model biofilm-forming pathogenic microorganism and is considered to be the most threatening pathogen causing biofilm in a human host [3]. *P. aeruginosa* causes urinary tract infections, kidney infections, cystic fibrosis, surgical site infection and sepsis [3], [4], [5], [6]. Bacteria in biofilm form present different behaviors than do their planktonic forms [3]. Biofilm is a protected form of cell growth that permits bacteria to endure in aggressive conditions and also separate to inhabit novel niches [7]. Further, it is important to be aware of what makes biofilm growth distinct from planktonic growth, as it is vital to expanding therapeutic interventions to treat biofilm infections [8]. Eliminating biofilm usually requires higher and continued antibiotic doses, and this often does not successfully eradicate biofilm infections. In many cases, a combination of antimicrobial therapies is necessary to eradicate the biofilm infection [9]. Methods of producing synergy against biofilms primarily involve a combination of two antibiotics, as well as antibiotics with a considerable diversity of probable anti-biofilm compounds [10]. The aim of the present study was to evaluate *in vitro* the synergy between routinely used antibiotics for inhibition of the planktonic and biofilm forms of clinical isolates *P. aeruginosa*. 

## Materials and methods

**Bacterial isolates:** Thirty-six non-duplicated *P. aeruginosa* isolates were obtained from clinical specimens and identified by colony morphology, Gram staining and standard biochemical tests [11] at the Microbiology Department of Tabriz University of Medical Sciences during 2014–2015. 

**Antibiotic susceptibility testing:** Disk-diffusion susceptibility testing was performed according to the Clinical and Laboratory Standards Institute (CLSI) guidelines [12]. The antibiotic disks (MAST, England) used were aztreonam (30 µg), imipenem (10 µg), meropenem (10 µg), colistin (10 µg), amikacin (30 µg), cefepime (30 µg) ceftazidime (30 µg), tobramycin (30 µg), gentamicin (30 µg), ciprofloxacin (5 µg), polymyxin B (300 units), gatifloxacin (5 µg) and piperacillin/tazobactam (100/10 µg). The quality of susceptibility testing was validated using the American Type Culture Collection quality-control strain *P. aeruginosa* ATCC 27853 [13]. 

**Quantitative detection of biofilm:** The microtiter plate assay was employed to quantitatively detect biofilm. Three to five colonies were suspended in 5 mL of TSB and incubated for 18 h at 37°C without shaking. The stationary phase culture was vortexed and then diluted 1:100 in TSB with 1% glucose. 200 µL of this solution was incubated in 96 well plates for 18 h at 37ºC. Medium with suspended bacteria was then removed. The plates were carefully washed 4 times with water and air dried before staining with 200 µL of 0.9% crystal violet solution for 15 min. After removing the dye solution and washing with water, the attached dye was solubilized with 95% ethanol and the optical density of the adherent biofilm was determined twice by microtiter plate reader at OD of 450–630. In the present study, we used TSB containing 1% glucose as a negative control [14].

**MIC determination:** The MICs of colistin, ceftazidime, clarithromycin, amikacin, and tobramycin were determined by the broth microdilution technique using cation-adjusted Mueller-Hinton broth (CAMHB). Stock solutions, adjusted for potency, were prepared immediately prior to testing. The MICs were determined according to the CLSI guidelines for broth microdilution. The MIC was defined as the lowest concentration of antibiotic that completely inhibited the growth of the organism as detected by the unaided eye [13].

**FIC determination:** The antibacterial effects of combinations of amikacin plus ceftazidime, tobramycin plus colistin, tobramycin plus clarithromycin and ceftazidime plus colistin were detected with the checkerboard assay and determination of FICI (Fractional Inhibitory Concentration Index). For the checkerboard test, the MIC of each antibiotic was determined alone and in combinations against each isolate in one 96-well plate. Positive growth controls were performed in wells without antibiotic to check for the existence of turbidity. The concentration ranges of each antimicrobial agent in combination ranged from 1 to 32 times the MIC. Dilutions of drugs A and B were prepared with a twofold dilution. The FICI was determined as follows: 


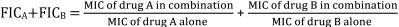


Synergy was defined as an FICI ≤0.5, additivity/indifference was defined as an FICI >0.5 to 4, and antagonism was defined as an FICI >4 [15], [16].

**BIC determination:** The biofilm inhibitory concentration (BIC) was determined to determine the antibiofilm activity of drugs. About 100 µg of microbial suspension equal to 0.5 McFarland in nutrient broth were transferred to the wells of a flat-bottomed 96-well microtiter plate. Biofilm formation was induced by dipping the pegs of a modified polystyrene microtiter lid into this biofilm growth plate and incubating at 37°C for 20 h. Peg lids were rinsed three times in sterile water, placed onto flat-bottomed microtiter plates containing serial concentrations of amikacin, tobramycin, colistin, ceftazidime and clarithromycin only and also in combination with each other in CAMHB per well, and incubated for 20 h at 37°C. The peg lids in sterile water placed into antibiotic-free CAMHB in a flat-bottomed microtiter plate. To transfer biofilms from the pegs to wells, each plate was centrifuged at 805 g for 20 min. The peg lid was changed by a usual cover. The Optic Density (OD) at 650 nm was determined on a microtiter plate colorimeter before and after incubation at 37°C for 6 h. The BIC was defined as the lowest concentration of an antimicrobial that lid in an OD650 variation at or below 10% of the mean of two positive control well readings [17].

## Results

**Figure 1:**
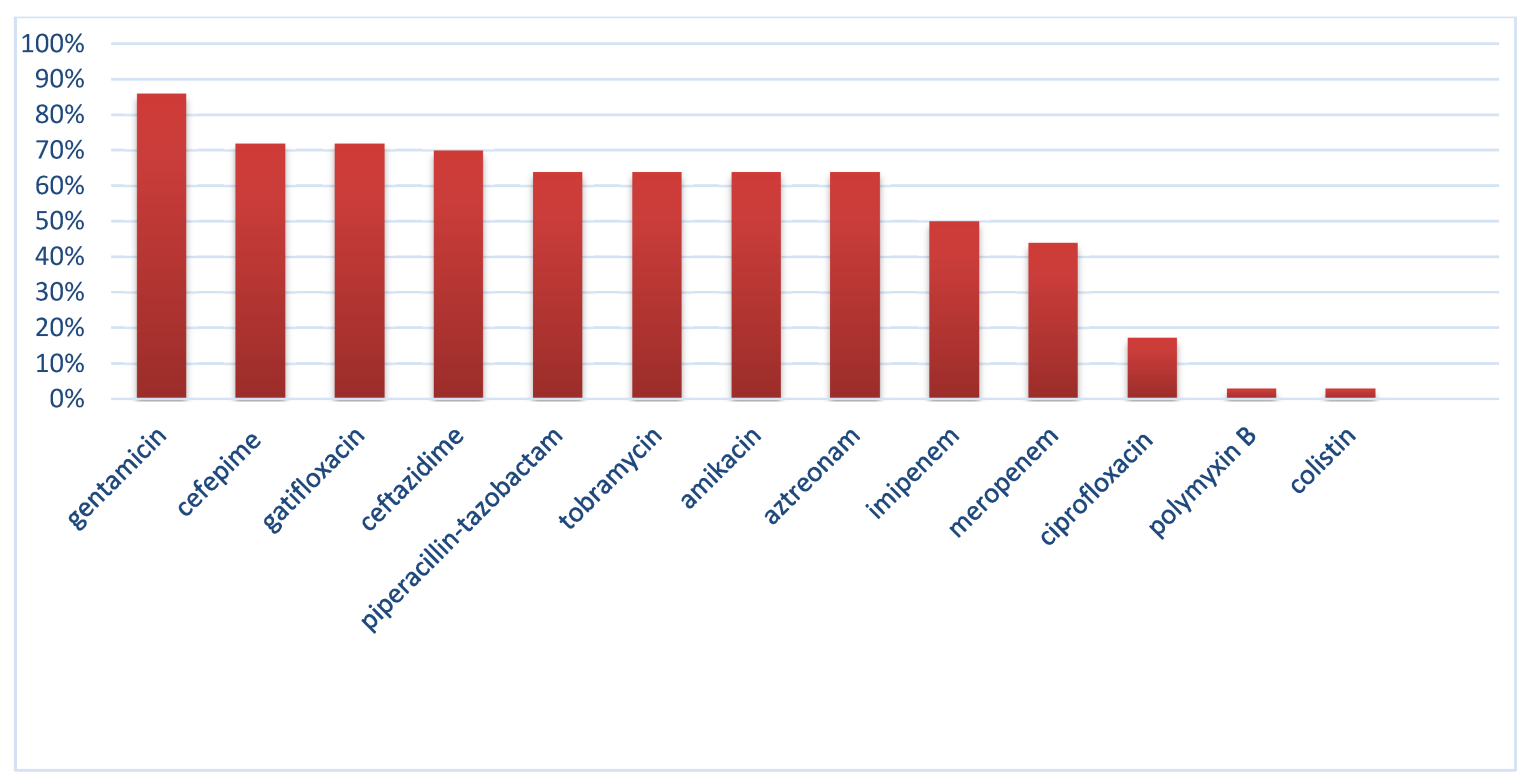
Antibiotic non-susceptibility patterns of bacterial isolates

We evaluated 36 clinical isolates of *Pseudomonas aeruginosa* obtained from different infections sources. The highest rate of resistance was against gentamicin, 86%, while the highest sensitivity rates were discovered for colistin and polymyxin. Resistance rates were 44.4% and 50% for meropenem and imipenem, respectively. A high frequency of resistance (above 60%) was observed for other tested antibiotics. Figure 1 (Fig. 1) displays the frequency of resistance to tested antibiotics. According to the disk diffusion method, 58.3% of isolates were MDR. According to the microbroth dilution assay, the range of colistin MIC was 0.5–4 µg/ml. The MIC_50_ and MIC_90_ of colistin were found to be 1 and 2 µg/ml, respectively. As expected, all isolates were resistant to clarithromycin (the MIC of isolates were 512 and 1024 µg/ml). The MIC ranges, MIC_50_ and MIC_90_ of other antibiotics are shown in Table 1 (Tab. 1). 

The synergetic effects between amikacin/ceftazidime, tobramycin/colistin and ceftazidime/colistin were observed in 55.6%, 58.3% and 52.8% of isolates, respectively. The FICI, FICI_50_ and FICI_90_ for each combination are presented in Table 2 (Tab. 2). 

**Figure 2:**
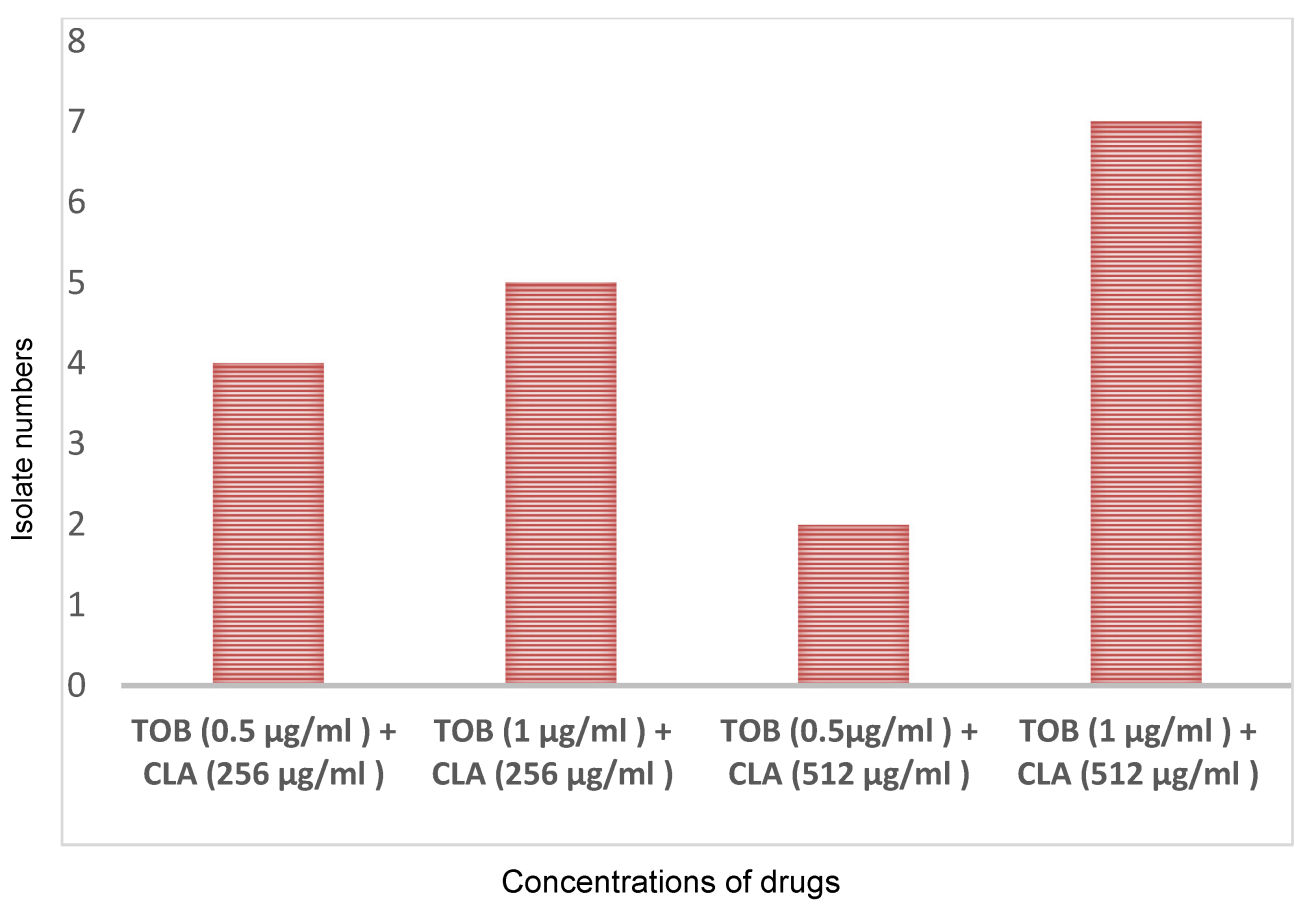
Antibiofilm effects of the combination of tobramycin (TOB) and clarithromycin (CLA)

According to the microtiter plate assay, 26 isolates (72.2%) were biofilm producers. Of these, 2 isolates (5.6%) were strong biofilm producers; the frequency of moderate and weak biofilm-producing isolates was 22.2% (8 isolates) and 44.4% (16 isolates), respectively. The antibiofilm effect of antibiotics was tested alone or in combinations against biofilm-producing isolates. A synergetic effect was found between tobramycin and clarithromycin on the preformed biofilm. However, tested individually, amikacin, tobramycin, colistin, ceftazidime, and clarithromycin did not show a significant antibiofilm effect. Although clarithromycin alone did not show any significant effect at a high concentration on the planktonic or biofilm form, we observed considerable synergetic activity when the biofilm was co-treated with tobramycin at 0.5–1 µg/ml and clarithromycin at a concentration of 256–512 µg/ml. This combination in these concentrations produced an OD650 difference at or below 10% of the mean of two positive controls. This effect observed in 18 of 26 (69.2%) biofilm-producing isolates (Figure 2 (Fig. 2)). Clarithromycin in combination with amikacin, colistin or ceftazidime did not show an antibiofilm effect. Amikacin/colistin, amikacin/ceftazidime, tobramycin/colistin and tobramycin/ceftazidime were ineffective on biofilm. 

## Discussion

Hospital-acquired infections caused by *P. aeruginosa* are generally life-threatening and pose a great challenging to treat. Selection for resistant strains during antimicrobial therapy among initially susceptible isolates often happens with this organism, resulting in the emergence of multidrug resistance especially in health care settings [18]. In the present study, similar to other studies carried out by Memar et al. (2016) and Gill et al. (2011), a high frequency of resistance was found to β-lactams, aminoglycosides, and quinolones [19], [20]. An emergence of MDR strains decreases the effectiveness of routine antibiotics in empirical therapy. In this study, 58.3% of tested isolates were identified as MDR and unsusceptible to at least one agent in three or more antimicrobial categories. Different prevalences of MDR *P. aeruginosa* (from 20% to 100%) have been reported by others researchers [21], [22]. Some factors, such as geographic diversity, patient’s demographical factors, or access and exposure to antimicrobial agents play important roles in the frequency and acquisition of MDR *P. aeruginosa* isolates [19]. The range of effective therapeutic options has become severely limited in recent years, in healthcare most commonly for MDR *P. aeruginosa* isolates. Combination therapy is one of the most effective strategies for managing this problem. Therefore, a widespread interest exists in administrating conventional antibiotics in combination in order to enhance antimicrobial effects and improve patient condition. Combination therapy may expand the antibacterial spectrum, prevent the spread of resistant isolates, decrease side effects and offer a synergetic effect. Synergy testing has shown evidence of an interaction of two antibiotics in combination against bacterial isolates [23]. A synergetic effect between amikacin/ceftazidime, tobramycin/colistin and ceftazidime/colistin was observed in 55.6%, 58.3% and 52.8% of isolates, respectively. Similar to a study by Gunderson et al. that reported a synergetic effect between ceftazidime and colistin, we observed significant synergetic effects of this combination in 52.8% of isolates [24]. In agreement with our results, Berlana et al. reported that patients treated with both colistin and tobramycin had a shorter duration of hospitalization and fewer periods of antibiotic administration than patients treated with one of these drugs alone [25]. Other researchers have reported a synergetic effect between colistin and other antibiotics, such as rifampin and carbapenems [26], [27]. Due to its significant *in vitro* antibacterial effects against MDR *P. aeruginosa*, colistin is frequently the only therapeutic option appropriate for the treatment of infections with this pathogen; therefore, its use has increased significantly in recent years, especially in hospital-acquired infections [19], [27]. Unfortunately, increasing the daily dose may not be a good choice, because nephrotoxicity is a dose-limiting side effect and arises in 30 to 50% of cases. It is consequently not surprising that suboptimal applications incite the development of resistance to colistin, which seriously limits colistin therapy. *In vivo* and *in vitro* evaluation yielded evidence of the potential for the rapid development of colistin resistance during mono-therapy [27]. This study showed that the combination of colistin with an anti-pseudomonas agent such as ceftazidime or amikacin enhances the antibacterial effect against MDR *P. aeruginosa* [19]. *P. aeruginosa* can form biofilms, which could significantly inhibit its eradication during antibiotic therapy and stimulate recurrent infections. Conventional antibiotic susceptibility testing surveys the efficiency of antibiotics against the planktonic form of organisms under aerobic conditions [28]. Thus, the determination of an antibiotic’s BIC may be advantageous in the treatment of infections with biofilm-producing *P. aeruginosa*. Clarithromycin has been reported to provide potential inhibition of *P. aeruginosa* biofilm with a decrease in bacterial virulence factor expression when used at sub-MIC [29]. We detected a synergetic effect between tobramycin and clarithromycin on the preformed biofilm. Lutz et al. reported a macrolide decrease at the MIC of other antimicrobial agents against *P. aeruginosa* in a biofilm. Hardy et al. reported synergetic activity for the combination of tobramycin (0.2 µg/ml) and clarithromycin (300 µg/mL, 400 µg/mL and 500 µg/mL) against biofilm [30]. 

In conclusion, *P. aeruginosa* clinical isolates are highly susceptible to the combination of antibiotics. The administration of these combinations may enhance the antibacterial and antibiofilm activity of conventional antibiotics. Further investigations of isolates in a clinical setting are necessary to assess the efficacy of these combinations against *P. aeruginosa* infections. The results of this study show that the combination of antibiotics has different effects on biofilm and planktonic forms. Therefore, separate detection of antibacterial and antibiofilm effects of the antibiotic in the combinations is useful for guiding antibiotic therapy. 

## Notes

### Acknowledgements

This project was financially supported by the Tropical and Infectious Diseases Research Center, Tabriz University of Medical Sciences. This article was written based on the dataset of an M. Sc. thesis, registered at Tabriz University of Medical Sciences. 

### Competing interests

The authors declare that they have no competing interests.

## Figures and Tables

**Table 1:**
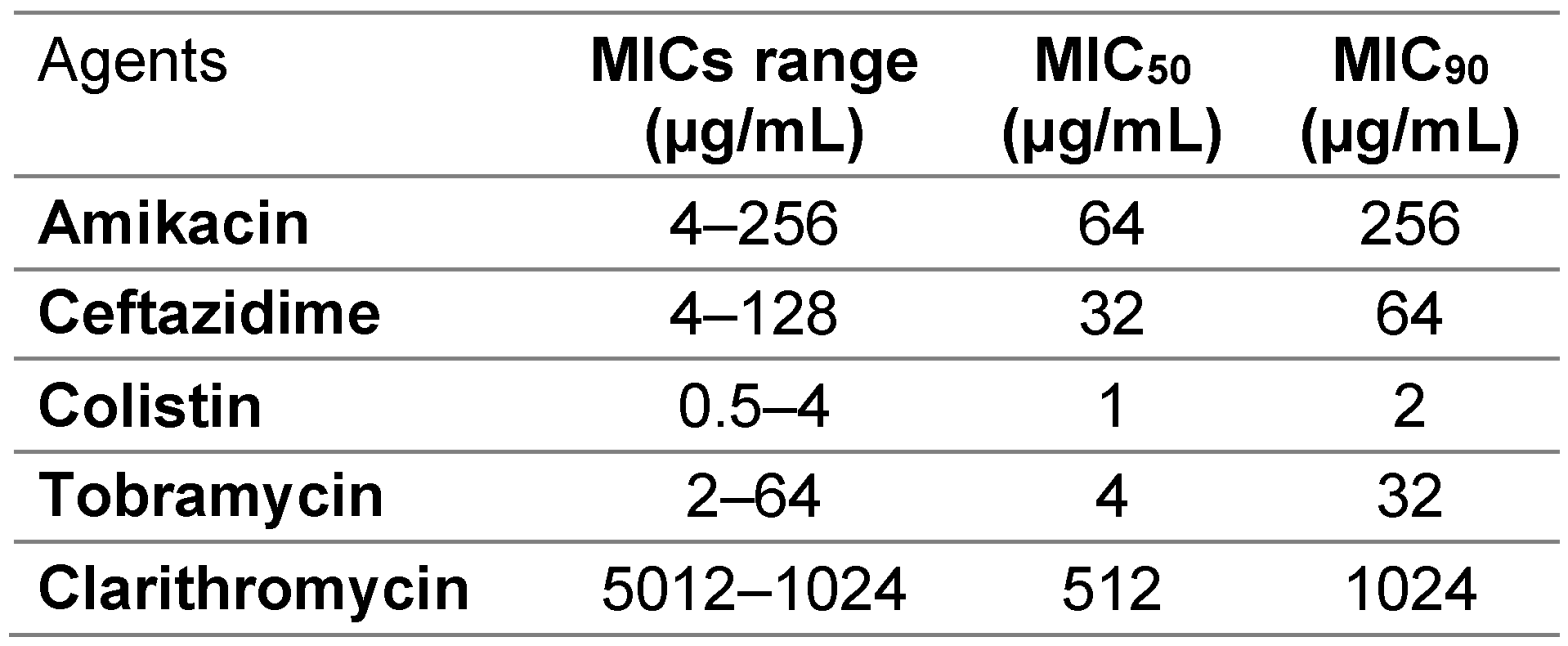
The MIC ranges, MIC_50_, and MIC_90_ of antibiotics against the planktonic form of isolates

**Table 2:**
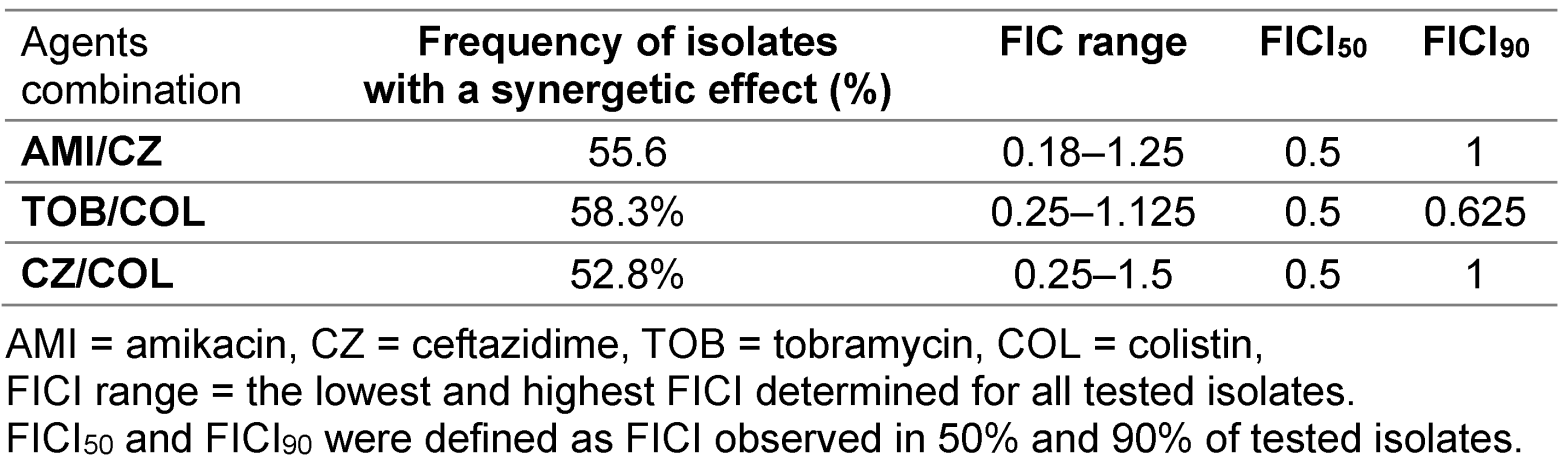
The FIC ranges, FICI_50_, and FICI_90_ of antibiotic combinations against the planktonic form of isolates

## References

[1] Perez-Jorge C, Gomez-Barrena E, Horcajada JP, Puig-Verdie L, Esteban J (2016). Drug treatments for prosthetic joint infections in the era of multidrug resistance. Expert Opin Pharmacother.

[2] Velez Perez AL, Schmidt-Malan SM, Kohner PC, Karau MJ, Greenwood-Quaintance KE, Patel R (2016). In vitro activity of ceftolozane/tazobactam against clinical isolates of Pseudomonas aeruginosa in the planktonic and biofilm states. Diagn Microbiol Infect Dis.

[3] Das MC, Sandhu P, Gupta P, Rudrapaul P, De UC, Tribedi P, Akhter Y, Bhattacharjee S (2016). Attenuation of Pseudomonas aeruginosa biofilm formation by Vitexin: A combinatorial study with azithromycin and gentamicin. Sci Rep.

[4] Akhi MT, Ghotaslou R, Beheshtirouy S, Asgharzadeh M, Pirzadeh T, Asghari B, Alizadeh N, Toloue Ostadgavahi A, Sorayaei Somesaraei V, Memar MY (2015). Antibiotic Susceptibility Pattern of Aerobic and Anaerobic Bacteria Isolated From Surgical Site Infection of Hospitalized Patients. Jundishapur J Microbiol.

[5] Vitkauskienė A, Skrodenienė E, Dambrauskienė A, Macas A, Sakalauskas R (2010). Pseudomonas aeruginosa bacteremia: resistance to antibiotics, risk factors, and patient mortality. Medicina (Kaunas).

[6] Cole SJ, Lee VT (2015). Cyclic Di-GMP Signaling Contributes to Pseudomonas aeruginosa-Mediated Catheter-Associated Urinary Tract Infection. J Bacteriol.

[7] Hall-Stoodley L, Costerton JW, Stoodley P (2004). Bacterial biofilms: from the natural environment to infectious diseases. Nat Rev Microbiol.

[8] Mulcahy LR, Isabella VM, Lewis K (2014). Pseudomonas aeruginosa biofilms in disease. Microb Ecol.

[9] Wu H, Moser C, Wang HZ, Høiby N, Song ZJ (2015). Strategies for combating bacterial biofilm infections. Int J Oral Sci.

[10] Ribeiro SM, Felício MR, Boas EV, Gonçalves S, Costa FF, Samy RP, Santos NC, Franco OL (2016). New frontiers for anti-biofilm drug development. Pharmacol Ther.

[11] Mahon CR, Manuselis G (2000). Textbook of diagnostic microbiology.

[12] Clinical and Laboratory Standards Institute (2011). Performance Standards for Antimicrobial Susceptibility Testing: Twenty First Informational Supplement M100-S21.

[13] Clinical and Laboratory Standards Institute (2014). Performance Standards for Antimicrobial Susceptibility Testing: Twenty Fourth Informational Supplement M100-S24.

[14] Hassan A, Usman J, Kaleem F, Omair M, Khalid A, Iqbal M (2011). Evaluation of different detection methods of biofilm formation in the clinical isolates. Braz J Infect Dis.

[15] Bonapace CR, Bosso JA, Friedrich LV, White RL (2002). Comparison of methods of interpretation of checkerboard synergy testing. Diagn Microbiol Infect Dis.

[16] Hall MJ, Middleton RF, Westmacott D (1983). The fractional inhibitory concentration (FIC) index as a measure of synergy. J Antimicrob Chemother.

[17] Moskowitz SM, Foster JM, Emerson J, Burns JL (2004). Clinically feasible biofilm susceptibility assay for isolates of Pseudomonas aeruginosa from patients with cystic fibrosis. J Clin Microbiol.

[18] Obritsch MD, Fish DN, MacLaren R, Jung R (2005). Nosocomial infections due to multidrug-resistant Pseudomonas aeruginosa: epidemiology and treatment options. Pharmacotherapy.

[19] Memar MY, Pormehrali R, Alizadeh N, Ghotaslou R, Bannazadeh Baghi H (2016). Colistin, an option for treatment of multiple drug resistant Pseudomonas aeruginosa. Physiology and Pharmacology.

[20] Gill MM, Usman J, Kaleem F, Hassan A, Khalid A, Anjum R, Fahim Q (2011). Frequency and antibiogram of multi-drug resistant Pseudomonas aeruginosa. J Coll Physicians Surg Pak.

[21] De Francesco MA, Ravizzola G, Peroni L, Bonfanti C, Manca N (2013). Prevalence of multidrug-resistant Acinetobacter baumannii and Pseudomonas aeruginosa in an Italian hospital. J Infect Public Health.

[22] Moazami-Goudarzi S, Eftekhar F (2013). Assessment of carbapenem susceptibility and multidrug-resistance in Pseudomonas aeruginosa burn isolates in Tehran. Jundishapur Journal of Microbiology.

[23] Saiman L (2007). Clinical utility of synergy testing for multidrug-resistant Pseudomonas aeruginosa isolated from patients with cystic fibrosis: 'the motion for'. Paediatr Respir Rev.

[24] Gunderson BW, Ibrahim KH, Hovde LB, Fromm TL, Reed MD, Rotschafer JC (2003). Synergistic activity of colistin and ceftazidime against multiantibiotic-resistant Pseudomonas aeruginosa in an in vitro pharmacodynamic model. Antimicrob Agents Chemother.

[25] Berlana D, Llop JM, Manresa F, Jódar R (2011). Outpatient treatment of Pseudomonas aeruginosa bronchial colonization with long-term inhaled colistin, tobramycin, or both in adults without cystic fibrosis. Pharmacotherapy.

[26] Hogg GM, Barr JG, Webb CH (1998). In-vitro activity of the combination of colistin and rifampicin against multidrug-resistant strains of Acinetobacter baumannii. J Antimicrob Chemother.

[27] Bergen PJ, Forrest A, Bulitta JB, Tsuji BT, Sidjabat HE, Paterson DL, Li J, Nation RL (2011). Clinically relevant plasma concentrations of colistin in combination with imipenem enhance pharmacodynamic activity against multidrug-resistant Pseudomonas aeruginosa at multiple inocula. Antimicrob Agents Chemother.

[28] Tré-Hardy M, Nagant C, El Manssouri N, Vanderbist F, Traore H, Vaneechoutte M, Dehaye JP (2010). Efficacy of the combination of tobramycin and a macrolide in an in vitro Pseudomonas aeruginosa mature biofilm model. Antimicrob Agents Chemother.

[29] Lutz L, Pereira DC, Paiva RM, Zavascki AP, Barth AL (2012). Macrolides decrease the minimal inhibitory concentration of anti-pseudomonal agents against Pseudomonas aeruginosa from cystic fibrosis patients in biofilm. BMC Microbiol.

[30] Tré-Hardy M, Vanderbist F, Traore H, Devleeschouwer MJ (2008). In vitro activity of antibiotic combinations against Pseudomonas aeruginosa biofilm and planktonic cultures. Int J Antimicrob Agents.

